# Use of Brilliant Blue G in Descemet's Membrane Endothelial Keratoplasty

**DOI:** 10.1155/2017/9720389

**Published:** 2017-06-06

**Authors:** Takahiko Hayashi, Kentaro Yuda, Itaru Oyakawa, Naoko Kato

**Affiliations:** ^1^Department of Ophthalmology, Yokohama Minami Kyosai Hospital, Yokohama, Japan; ^2^Department of Ophthalmology, Yokohama City University Hospital, Yokohama, Japan; ^3^Kikuna Yuda Eye Clinic, Yokohama, Japan; ^4^Department of Ophthalmology, Saitama Medical University Hospital, Saitama, Japan; ^5^Department of Ophthalmology, Heart Life Hospital, Okinawa, Japan; ^6^Department of Ophthalmology, Ryukyu University, Okinawa, Japan

## Abstract

Vital staining of the endothelial graft is essential during Descemet's membrane endothelial keratoplasty (DMEK) to ensure surgical success. DMEK surgeons worldwide commonly use trypan blue (TB) to this end. However, TB may exert toxic effects on both the cornea and retina. Recently, Brilliant Blue G (BBG) has become recognized as an alternative stain for use during vitreoretinal surgery; BBG is associated with lower levels of toxicity. We retrospectively analyzed the utility of BBG staining during DMEK. We used 0.1% (w/v) BBG to stain the DMEK grafts of 12 patients. We evaluated the best spectacle-corrected visual acuity (BSCVA), central corneal thickness (CCT), and endothelial cell density (ECD) before and 3 and 6 months after surgery. BBG was effective in terms of graft visualization during DMEK. The BSCVA (log  MAR) improved from 0.99 ± 0.57 to 0.01 ± 0.07 (*p* < 0.05). The CCT decreased from 720.3 ± 58.1 *μ*m preoperatively to 511.5 ± 50.6 *μ*m at 6 months postoperatively (*p* = 0.0001). The ECD decreased from 2,754 ± 296 cells/mm^2^ to 1,708 ± 426 cells/mm^2^ at 6 months postoperatively (*p* < 0.001). The ECD loss was 37.9 ± 16.3%. The outcomes using BBG were comparable to those of earlier reports that employed TB; thus, BBG may be a viable alternative to TB.

## 1. Introduction

Descemet's membrane endothelial keratoplasty (DMEK) is a valuable method of corneal transplantation that corrects corneal endothelial dysfunction [1, 2]. DMEK has recently become widely accepted; the procedure facilitates rapid visual recovery [3, 4]. The advantages of DMEK include good visual outcomes and low-level immunological rejection, but DMEK is associated with a steep surgical learning curve. DMEK is difficult in terms of graft preparation, orientation, insertion, and unfolding [5–10].

Good visualization of the DMEK graft is important; adequate graft staining facilitates successful surgery. Misdiagnosis of graft orientation causes both inverted graft attachment and primary graft failure [11]. Vital dye staining facilitates the identification of graft orientation. Various methods have been used to determine graft orientation, including graft staining per se [12], graft marking [13], graft stamping [14], and intraoperative optical coherence tomography (OCT) [15, 16]. We have trialled all of these methods. We find that adequate staining of the DMEK graft is indispensable in terms of appropriate graft orientation; graft identification is difficult when the color fades. Almost all DMEK surgeons use the trypan blue (TB) stain to this end; commercial preparations include VisionBlue® (Dutch Ophthalmic Research Center, Zuidland, Netherlands) and MembraneBlue® (Dutch Ophthalmic Research Center) [12].

Vital stains do not kill living cells. Vital dyes have been used both diagnostically and surgically by various specialists, including oncologists, when performing cataract and vitreoretinal surgery. TB is employed in the course of cataract surgery to visualize the anterior capsule during capsulorhexis [17, 18]. During vitrectomy, indocyanine green (ICG) [19] and Brilliant Blue G (BBG) [20] are used to stain the internal limiting membrane, whereas both TB [21] and triamcinolone acetonide [22] aid in the identification of epiretinal membranes. Both TB and ICG have been employed to stain the anterior capsule [23, 24]. TB is minimally toxic to the corneal endothelium [25]; however, both ICG and TB have been associated with retinal cell toxicity [26, 27]. In contrast, BBG is less toxic to corneal endothelial cells [28], although the dye has more commonly been used to stain the inner limiting membrane of the retina during vitreoretinal surgery [29]. However, recent reports have suggested that long-term, high-level TB exposure is associated with increased toxicity and decreases the endothelial cell density (ECD) [12]. Thus, the toxicities of vital dyes used during DMEK should be considered.

In the present study, we analyzed data from DMEK patients for whom BBG (i.e., not TB) was used to identify the grafts.

## 2. Materials and Methods

### 2.1. Donor Preparation

**Figure 1:**
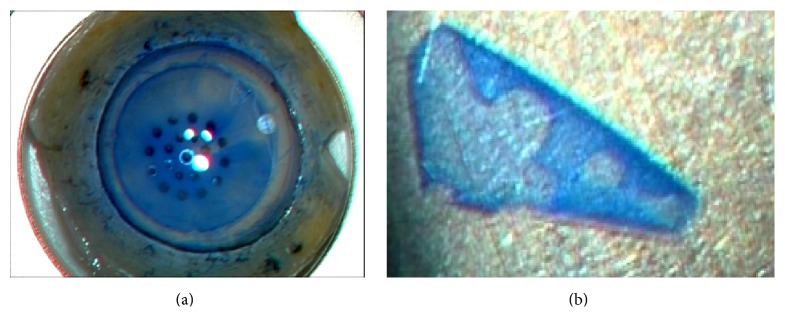
A photograph of a Descemet membrane graft in preparation. (a) BBG (0.1% [w/v]) is applied when preparing the graft. The edge of the graft is well-stained. (b) After peeling was concluded, we stained the graft with 0.1% (w/v) BBG for an additional 1 min. We preserved the graft in a dish replenished continuously with BSS for 30 min prior to insertion of the graft into the eye.

BBG 250® (BBG; Sigma-Aldrich, St. Louis, MO, USA) was dissolved in balanced saline solution (BSS or BSS-plus; Alcon, Osaka, Japan) to 0.1% (w/v). All dye osmolarities were ca. 298 mOsm, and the pH values were 7.4. We used donor tissue from SightLife (Seattle, WA, USA) for DMEK. All grafts were peeled as described previously. BBG (0.1%, w/v) was used to stain the graft edges during peeling (Figure 1). A punch was gently placed on the endothelial surface to indent a circle 7.75 or 8.0 mm in diameter. Next, 1.0 and 1.5 mm diameter dermatological biopsy punches (Kai Industries, Seki, Japan) were used to place asymmetric marks on the edges of the identified circles. Donor grafts thus marked were stained with 0.1% (w/v) BBG (1.0 mg/mL) for 1 min and stored in BSS prior to insertion 30 min later.

### 2.2. Patients

Our study protocol was approved by the Institutional Review Board of Yokohama Minami Kyosai Hospital (approval number 26_1_2). Written informed consent was obtained from all patients prior to enrolment in the study. We carefully followed all ethical principles of the Declaration of Helsinki. Twelve eyes of 12 patients with bullous keratopathy who underwent DMEK at Yokohama Minami Kyosai Hospital from February 2016 to August 2016 and who were followed up for more than 6 months were retrospectively analyzed. We treated 2 males and 10 females of mean age 75.9 ± 4.5 years. All eyes underwent DMEK performed by a single surgeon (TH). Eight eyes exhibited iatrogenic bullous keratopathy; five had undergone prior argon laser iridotomy (ALI) and three prior cataract surgery and intraocular lens implantation. Two eyes exhibited Fuchs' corneal endothelial dystrophy and two corneal endotheliopathy attributable to pseudoexfoliation syndrome (PEX).

### 2.3. Surgical Techniques and Postoperative Treatment

All surgeries were performed under local anesthesia. After establishing retrobulbar anesthesia and a Nadbath facial nerve block, 2 paracenteses and a 2.8 mm upper corneal or corneoscleral incision were made for the recipient cornea. Peripheral iridotomy was performed at the 6-o'clock position using a 25-gauge vitreous cutter to prevent the development of a postoperative pupillary block. The donor membrane graft stained with 0.1% (w/v) BBG (1.0 mg/ml) was placed into an intraocular lens injector (model WJ-60M; Santen Pharmaceuticals, Osaka, Japan) and inserted into the anterior chamber. A small amount of air was also injected between the host cornea and donor graft, and the rolled-up donor graft was then unfolded. Correct graft orientation was confirmed with reference to the preoperative marks. The anterior chamber was filled with air to allow the graft to adhere to the host cornea. Fifteen minutes later, the air was partially replaced with BSS. Finally, 0.4 mg of betamethasone (Rinderon®; Shionogi, Osaka, Japan) was subconjunctivally administered in 1.5% (w/v) levofloxacin eyedrops (Cravit®; Santen Pharmaceuticals).

Postoperative medications included 1.5% (w/v) levofloxacin (Cravit®), 0.1% (w/v) betamethasone sodium phosphate (Sanbetasone®; Santen Pharmaceuticals), and 2% (w/v) rebamipide ophthalmic solution (Mucosta®; Otsuka, Tokyo, Japan), commencing at four times daily for 3 months and tapering thereafter.

### 2.4. Examinations

In addition to the standard ophthalmic examination, all of the best spectacle-corrected visual acuity (BSCVA), corneal ECD, central corneal thickness (CCT), and graft adaptation were evaluated both preoperatively and for up to 6 months postoperatively. Graft adaptation was assessed with the aid of both slit-lamp biomicroscopy and anterior segment OCT (SS1000 instrument; Tomey, Nagoya, Japan). Corneal thickness was measured via corneal tomography (SS1000; Tomey). Preoperative ECDs were retrieved from donor eye bank records. Intraoperative and postoperative complications were recorded and postoperative ECDs were measured with the aid of a specular microscope (model FA3509; Konan Medical, Nishinomiya, Japan).

### 2.5. BBG Staining Capacity

The BBG staining capacity was evaluated by two experienced ophthalmologists (TH and KY) as follows: W, worse than TB; S, similar to TB; and B, better than TB.

### 2.6. Statistical Analysis

The Wilcoxon test was used, when appropriate, to compare means among groups. All analyses were performed with the aid of StatView statistical software (Abacus Concepts, Berkeley, CA, USA). A *p* value < 0.05 was considered to reflect statistical significance.

## 3. Results

**Figure 2:**
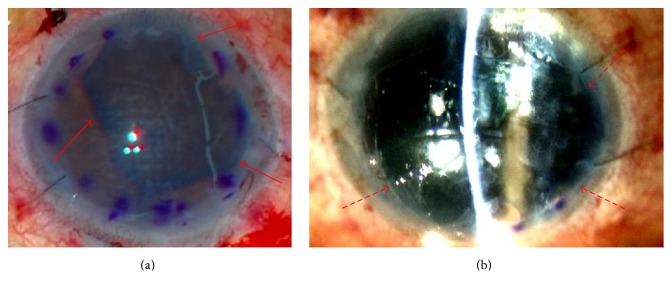
Photographs of a Descemet membrane graft during and after surgery. (a) An intraoperative photograph clearly reveals the Descemet membrane graft (red arrows). (b) Immediately after surgery, a well-stained graft is clearly visible on slit-lamp examination (broken red arrows).

Detailed patient profiles are shown in Table 1. Graft staining with BBG ensured adequate identification during surgery (Figure 2(a)). Donor graft orientation was apparent even when the graft exhibited strong corneal edema, ensuring that all grafts were appropriately inserted. Implanted donor grafts were clearly visible a few hours after surgery, and correct orientation was verified by slit-lamp biomicroscopy (Figure 2(b)).

The BSCVA (log MAR) improved significantly from 0.99 ± 0.57 preoperatively to 0.01 ± 0.07 at 6 months postoperatively (*p* = 0.001). The CCT decreased from 720.3 ± 58.1 *μ*m preoperatively to 511.5 ± 50.6 *μ*m at 6 months postoperatively (*p* = 0.0001). The corneal ECD was 1,708 ± 426 cells/mm^2^ at 6 months postoperatively (37.9 ± 16.3% less than the preoperative value of the donor graft). No eye showed any sign of pupillary blockage, microbial infection, or endothelial rejection. Partial graft detachment requiring rebubbling of the anterior chamber was observed in only one eye 6 days after surgery; the graft attained complete reattachment after rebubbling. No significant intraocular pressure (IOP) elevation was detected during the follow-up period. No upside-down insertion was noted. Notably, we encountered no instance of primary graft failure.

## 4. Discussion

Clinically, TB used during either cataract surgery or DMEK exhibits low toxicity. Indeed, we found, in a preliminary study, that neither TB nor BBG was toxic in terms of corneal ECD reduction after cataract surgery. In our preliminary work, the corneal endothelial cell losses 6 months after surgery were −2.2 ± 6.5% and −3.5 ± 9.8% in the TB and BBG groups, respectively (*p* = 0.737 [not significant, NS]; in our previous data).

However, after DMEK, the ECD loss was greater than that after cataract surgery, attaining 30–54% at 6 months postoperatively [5, 6, 30, 31]. This reflected extensive cell death, potentially attributable to not only surgical stress. We speculate that toxicity attributable to vital dyes may be in play. Although TB is widely used during DMEK, we suggest that the dye may be toxic after only brief contact with corneal endothelial cells. It is essential to maximally reduce any possible toxic effect. Vital dyes that actually protect the corneal endothelium must be identified. An in vivo study featuring anterior capsular staining showed that both ICG and TB triggered apoptosis in pig corneal endothelial cells, but BBG did not [28].

We make two useful points in the present study. First, 0.1% (w/v) BBG was not inferior to TB in terms of graft staining during DMEK. We noted no upside-down insertion of the DMEK graft. A recent report found that a lutein-based Brilliant Blue dye (lutein/zeaxanthin combined with Brilliant Blue [LZ/BB]), which is pale green in color, was valuable during both transplantation and graft preparation [32]. In the present study, we did not employ LZ/BB but rather BBG alone (which yields a dark blue color); both the staining capacity and graft visibility were similar to those afforded by TB (Table 1). Also, the endothelial cell loss after DMEK employing BBG staining was 37.9 ± 16.3% 6 months postoperatively, similar to what was reported earlier using TB staining. We earlier found that the ECD 6 months after DMEK employing TB staining was 1,273 ± 227 cells/mm^2^ during our learning curve. This ECD was lower than that associated with BBG staining, which might be attributed to learning curve or some other factors. Although postoperative endothelial cell loss is greatly affected by the surgical technique used (and the associated learning curve), we earlier found that a severe ECD reduction was associated with long-term TB use.

Our work has three principal limitations. First, this was an in vivo study. We found that ECD decreased significantly after DMEK, as have earlier reports that employed TB staining. An in vitro study is required to determine if ECD loss is indeed influenced by the chosen surgical procedure, BBG staining, or both. Additional in vitro experiments are required to understand why the ECD falls after DMEK. Also, we did not prospectively compare DMEK outcomes after the use of BBG or TB. Ideally, the two vital dyes should be employed (during DMEK) in a prospective randomized fashion. Our work was retrospective and lacks a formal comparison of the two dyes; we switched from TB to BBG during the time course of our series. Finally, we applied 0.1% (w/v) BBG for an arbitrary 1 min; the dye concentration upon dilution with BSS remains unknown. Further work is essential to validate the safety of DMEK facilitated by BBG staining. Higher TB concentrations afford excellent staining without adverse effects, but only if staining persists for 1–3 min; a longer staining time (5 min) reduced the ECD [12].

## 5. Conclusion

In conclusion, BBG is both efficacious and safe when used during DMEK. Additional studies are required in ocular surface surgeries, as with vitreoretinal surgeries.

## Figures and Tables

**Table 1:** Patient data.

Case	Sex	Age (years)	OD/OS	Etiology of BK	Preop. BSCVA	Preop. CCT (*μ*m)	Postop. BSCVA	Postop. CCT (*μ*m)	Staining capacity
(1)	F	71	OS	LI	20/60	712	20/15	565	S
(2)	M	79	OS	PEX	20/400	737	20/30	598	S
(3)	F	74	OD	PEX	20/60	698	20/25	531	S
(4)	M	69	OS	PBK	20/30	604	20/15	456	S
(5)	F	73	OD	FCED	20/400	744	20/25	446	S
(6)	F	78	OD	LI	20/100	763	20/15	473	S
(7)	F	81	OS	LI	20/1000	652	20/20	520	S
(8)	F	78	OS	LI	20/60	771	20/20	570	S
(9)	F	70	OD	FCED	20/60	744	20/20	491	S
(10)	F	83	OD	PBK	20/600	708	20/20	512	S
(11)	F	74	OS	LI	20/2000	834	20/25	510	S
(12)	F	81	OD	PBK	20/400	676	20/20	509	S

OD, right eye; OS, left eye; preop., preoperative; postop., postoperative; BSCVA, best-corrected scale-corrected visual acuity; CCT, central corneal thickness; BK, bullous keratopathy; PEX, pseudoexfoliation syndrome; FCED, Fuchs' corneal endothelial dystrophy; LI, laser iridectomy; staining capacity was scored as follows: W, worse than trypan blue (TB); S, similar to TB; and B, better than TB.
